# Multi-Target Behavior and Intent Prediction Under Incomplete Perception

**DOI:** 10.3390/s26175378

**Published:** 2026-08-25

**Authors:** Yongjie Ma, Yu Han, Xiaxin Zhang, Peng Ping

**Affiliations:** 1 School of Yonyou Digital and Intelligence, Nantong Institute of Technology, No. 211 Yongxing Road, Chongchuan District, Nantong 226005, China; 20239192@ntit.edu.cn; 2 School of Transportation and Civil Engineering, Nantong University, No. 9 Seyuan Road, Chongchuan District, Nantong 226019, China; 2433320018@stmail.ntu.edu.cn (Y.H.); 2433320001@stmail.ntu.edu.cn (X.Z.)

**Keywords:** intent prediction, situational awareness, particle filter, gated recurrent unit, threat field

## Abstract

**Highlights:**

**What are the main findings?**

- Proposed a TF-GRU model for multi-target intent prediction.
- Constructed static and dynamic threat fields to quantify risks.

**What are the implications of the main findings?**

- PF-DTW mechanism repairs missing data via adaptive switching.
- Cooperative particle filtering captures multi-target interactions.
- Achieved superior accuracy under incomplete perception conditions.

**Abstract:**

Predicting target intent in complex, dynamic multi-agent environments remains a formidable challenge due to incomplete perception and the highly dynamic nature of multi-target interactions. Conventional approaches—such as D-S evidence theory, expert systems, and Recurrent Neural Networks (RNNs)—are often constrained by data incompleteness and rigid behavioral assumptions, limiting their adaptability to dynamic high-value target identification and multi-target situational awareness on the ground. To address these challenges, a novel framework termed Threat Field–Gated Recurrent Unit (TF-GRU) is proposed. The TF-GRU framework integrates threat field modeling with a dynamic repair mechanism to enhance intent prediction under partial perception. Specifically, threat field modeling associates target attributes with intentions through the construction of static and dynamic threat fields, effectively capturing the temporal and semantic relationships among multiple targets. A particle filtering and dynamic time warping fusion strategy (PF-DTW) is employed to repair data gaps via short-term filtering and long-term trajectory matching, further refined by a neighborhood-angle constraint for accurate multi-target state estimation. In addition, trajectory and threat field features are processed using a Mish activation function and a threat-adaptive gating mechanism, which dynamically regulate information flow within the recurrent unit to model behavioral evolution. Experimental evaluations demonstrate that TF-GRU significantly enhances intent prediction accuracy under incomplete data conditions, thereby improving comprehensive situational awareness and supporting high-confidence decision-making in dynamic multi-target scenarios.

## 1. Introduction

In the increasingly complex operational environments of intelligent multi-agent systems—such as autonomous traffic management, drone fleet coordination, and smart logistics—situational awareness serves as a fundamental pillar of operational decision-making. It is defined as “the perception, comprehension, and projection of the future status of situational elements within a specific spatiotemporal context” [1,2]. At its core, situational awareness involves the integration of multi-source information to enable real-time understanding of the active environment, the spatial distribution of dynamic agents, and ongoing environmental changes. By inferring agent intentions, it provides anticipatory intelligence that supports proactive and adaptive control decisions. As a critical component of situational awareness [3,4,5], intent prediction focuses on inferring operational objectives through the analysis of behavioral patterns, trajectories, and deployment dynamics, thereby directly influencing both the depth of understanding and the accuracy of situational forecasting.

However, the complexity, dynamism, deceptiveness, and uncertainty of information in complex environments [6] present multiple challenges for intent prediction. First, information incompleteness remains a critical obstacle. Recent studies, such as Fang et al. [7] and Chen et al. [8], have addressed air combat intention recognition under incomplete information using fuzzy clustering and GRU-based models, highlighting the need for robust prediction in complex environments. Unmanned Aerial Vehicles (UAVs) and other sensors are highly susceptible to interference from smoke and fire, environmental occlusion, and target camouflage, which can cause data loss or introduce noise, thereby degrading the accuracy of behavioral pattern recognition and intent inference. Second, the unpredictable and adaptive nature of adversary behavior further complicates perception and prediction, especially in high-tempo confrontations where targets can rapidly adjust strategies and multi-entity coordination exacerbates the difficulty of evaluating overall tactical objectives. Moreover, the noisy and uncertain nature of sensory data requires situational awareness systems to distinguish between valid signals and environmental interference and to project future developments within limited timeframes, thereby imposing stringent demands on real-time responsiveness, adaptability, and robustness. In data-driven autonomous systems, rapid and accurate situational awareness is fundamental to ensuring navigation safety and optimizing multi-agent coordination. Therefore, the ultimate application purpose of the proposed intent prediction framework is to transform passive trajectory tracking into proactive situational awareness. By accurately anticipating adversary objectives, it enables autonomous ground systems to allocate resources, initiate countermeasures, and make high-confidence tactical decisions in advance.

In recent years, research on complex environment situational awareness and intent prediction has received increasing attention, with scholars investigating the topic from multiple theoretical perspectives. Studies have explored approaches such as deep learning-based temporal modeling, probabilistic reasoning, and multi-agent coordination, aiming to enhance the real-time understanding of complex environment dynamics and the inference of adversary intentions to support decision-making. However, several theoretical limitations remain. Most existing approaches primarily focus on single-target scenarios, thereby overlooking the complexities of multi-target coordination. Moreover, many models assume data completeness, which undermines robustness in information-scarce environments, and rely heavily on extensive prior knowledge, limiting adaptability to rapidly evolving complex environment conditions. These limitations underscore the necessity for more flexible and comprehensive frameworks capable of addressing incomplete perception and dynamic multi-target interactions. Detailed methodologies and specific advancements will be further elaborated in the following Section 2.

To improve target intent prediction under incomplete information, this study proposes a Gated Recurrent Unit-based prediction model (TF-GRU) that integrates threat field modeling with a dynamic data repair mechanism. The main contributions are as follows:

- Multi-dimensional threat quantification: By constructing both static and dynamic threat fields, the model quantifies target threat levels and explicitly links target characteristics to intentions, overcoming the semantic limitations of trajectory-only approaches.
- Adaptive repair via PF-DTW switching: A dynamic switching strategy between particle filter (PF) and dynamic time warping (DTW) is developed, adaptively selecting the repair method based on the duration of data absence, thereby enhancing robustness in incomplete information environments.
- Cooperative filtering for multi-target scenarios: Neighborhood target angle constraints are incorporated into cooperative particle filtering, improving collective behavior prediction in multi-target contexts.
- Extensive experimental results and theoretical analysis consistently demonstrate that the proposed TF-GRU model outperforms existing advanced techniques, highlighting its superior accuracy and robustness in intent prediction under incomplete information scenarios.

## 2. Related Works

The modern environment is increasingly complex, where incomplete information in multi-target scenarios complicates intent prediction and exposes the limits of traditional methods. Recent research addresses this challenge from three perspectives: target behavior modeling, multi-target collaborative inference, and missing-data handling. This paper systematically reviews and analyzes existing work in these areas.

### 2.1. Target Behavior Modeling and Multi-Target Collaborative Methods

As a core component of situational awareness, target intent recognition aims to infer potential tactical objectives through modeling of target kinematics and behavioral patterns. Early rule-driven approaches, including expert systems, template matching, and knowledge graphs [9,10], offered interpretability but exhibited limited generalizability in dynamic, multi-target environments. To enhance adaptability and autonomy, recent research has increasingly adopted data-driven methods, leveraging neural networks with strong sequential modeling capabilities to characterize target behaviors.

RNNs and their variants, such as Long Short-Term Memory (LSTM) and Gated Recurrent Unit (GRU), are widely used in behavior recognition and intent prediction. Lemin et al. [11] combined Bidirectional LSTM (BiLSTM) with Temporal Convolutional Networks (TCNs) to enhance recognition accuracy, while Kim et al. [12] employed a CNN-LSTM architecture for complex environment situational modeling. The Transformer, noted for global contextual modeling, has also been applied to trajectory prediction and intent inference [13], showing advantages in capturing long-range dependencies. In multi-target scenarios, Mohamed et al. [14] proposed an interaction modeling approach using Graph Convolutional Network (GCN) combined with trajectory clustering, significantly enhancing group motion prediction. Qi et al. [15] integrated graph structures with attention mechanisms, achieving effective coordination modeling for vehicles in traffic flow scenarios. Additionally, Foerster et al. [16] proposed a deep reinforcement learning-based multi-agent communication framework, enabling agents to learn negotiation and intent prediction, while Liu et al. [17] introduced the Graph Attention Isomorphism Network (GAIN) for long-term interaction modeling.

However, existing methods exhibit several limitations. First, research predominantly targets aerial or transportation domains, lacking specialized modeling for specialized ground agents constrained by terrain, mobility differences, and complex operational strategies. Second, most approaches assume complete behavioral data, overlooking information incompleteness due to sensor failure, occlusion, or interference [18]. Third, the absence of explicit modeling of operational semantic elements and multi-agent coordination impedes accurate linking of agent behaviors to high-level intent semantics. Additionally, these methods often overlook functional heterogeneity (e.g., influence radius, operational intensity, system resilience) and the influence of risk levels and agent importance in decision-making, as noted in dynamic Bayesian network frameworks [19].

### 2.2. Data Repair Methods Under Incomplete Perception Conditions

In complex environment, sensors often face occlusion, interference, or transmission failures, resulting in missing, delayed, or distorted observations. Recovering target states under incomplete perception is therefore essential for robust intent recognition.Particle filtering (PF), suitable for nonlinear and non-Gaussian systems, is widely applied to state estimation [20]. It represents the state space with sampled particles and updates their weights iteratively, enabling prediction despite missing data. Recent extensions [21,22,23,24] improve PF for network security situational awareness in military contexts, addressing particle degeneracy in prolonged gaps. However, PF suffers from particle degeneracy and drift when gaps persist. Dynamic time warping (DTW) aligns and compares time series by allowing nonlinear time scaling [25]. It preserves long-term patterns and trajectory shapes, supporting retrieval of historical behaviors that resemble current observations, thereby compensating for extended data loss. Yet DTW is less responsive to short-term dynamics and sensitive to noise without reliable reference trajectories. Deep generative models, such as Seq2Seq and GANs, have also been used to reconstruct missing sequences [26]. These methods depend on full training distributions and incur heavy computation, limiting their use in real-time complex scenarios. Chen et al. [27] proposed a graph attention network with spatial–temporal clustering to enhance traffic-related spatiotemporal feature representation and capture both recent-aware and periodic-aware dependencies. However, existing approaches still struggle to balance real-time recovery of short gaps with semantic consistency for long gaps, and seldom exploit inter-agent cooperation or collective intent propagation.

To address these gaps—namely, the lack of adaptive mechanisms for timely and semantically consistent data repair, insufficient modeling of ground-specific operational strategies, and limited use of multi-target coordination—this paper proposes TF-GRU, a GRU-based model integrating threat field modeling with a dynamic PF-DTW repair mechanism. By combining static and dynamic threat fields, PF-DTW fusion repair, and cooperative particle filtering, TF-GRU achieves high-precision recognition of ground multi-target intents under information-deficient conditions.

## 3. The Proposed Method: TF-GRU

To overcome limitations in robustness, coordination, and semantic representation, this paper proposes the TF-GRU model, which links target features to intentions via threat field modeling, repairs missing data with a dynamic PF-DTW mechanism, and captures multi-target interactions through cooperative particle filtering.

### 3.1. Problem Formulation and System Overview

The complex environment, with its deception, occlusion, and sensor limitations, causes noisy or missing data, while rapidly changing behaviors and coordinated target actions make intent prediction difficult, exposing the limits of traditional methods.

This paper proposes the TF-GRU model a GRU-based framework that integrates threat field modeling and dynamic data repair to enhance intent prediction accuracy and robustness. Static threat fields reflect spatial distribution based on target types such as tanks and artillery considering range firepower defense mobility and friendly enemy distance. Dynamic threat fields capture velocity acceleration orientation and temporal decay to model threat evolution.

**Figure 1:**
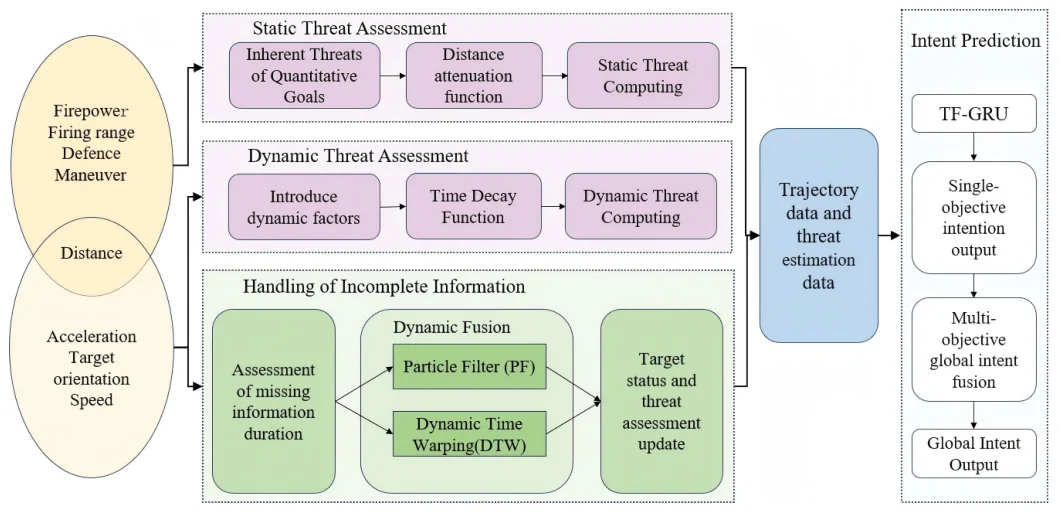
Overall framework of the proposed method.

To address missing data a PF-DTW switching mechanism combines PF with a constant acceleration model and cooperative neighborhood constraints for short-term gaps and DTW for long-term gap alignment. Weights adjust dynamically with missing duration balancing real-time tracking and pattern preservation. Repaired normalized trajectories fused with threat field features feed into an enhanced GRU using Mish activation adaptive Xavier initialization and threat-adaptive gating to capture temporal dynamics. Multi-target predictions weighted by importance ensure robust intent inference under incomplete data. The overall framework diagram is shown in Figure 1.

### 3.2. Threat Field Modeling

The primary challenge in static threat field modeling lies in the nonlinear coupling of multiple factors, heterogeneous target characteristics, and the tactical adaptability of spatial attenuation and weight allocation. This study focuses on tanks, armored vehicles, and artillery vehicles, selecting five key factors—friendly enemy distance *d*, range *l*, firepower *A*, defense *D*, and mobility *M*—to evaluate their impact on threat levels. Here, *d* is constant, *l* defines the effective attack range, *A* sets the upper limit of threat intensity, *D* enhances survivability and threat persistence, and *M* amplifies dynamic threat propagation via tactical positioning. Spatial attenuation characteristics of these factors are integrated and adapted to complex environment conditions. Threat intensity decay is governed by inherent attributes. A threat decay coefficient σ is introduced to quantify the attenuation of threat with distance.

For target types i∈{tank,armoredvehicle,artillery}, the static threat field is defined as

(1) $$W_{i}^{s}\left(x,y\right)=\left(A_{i}+D_{i}\right)\cdot M_{i}\cdot f_{i}\left(d,l_{i},\sigma_{i}\right)$$

where $f_{i}\left(d,l_{i},\sigma_{i}\right)$ is the distance attenuation function:

- Tank: The threat peaks at mid-range and diminishes at short and long distances:
  (2) $$f_{\mathrm{tank}}\left(d\right)=\exp\left(-\frac{{\left(d-l_{\mathrm{optimal}}\right)}^{2}}{2\sigma_{\mathrm{tank}}^{2}}\right)$$
- Armored vehicle: The threat decays rapidly with distance:
  (3) $$f_{\mathrm{armor}}\left(d\right)=\exp\left(-\frac{d}{\sigma_{\mathrm{armor}}}\right)$$
- Artillery vehicle: The threat is concentrated within the range, with high intensity inside and a sharp decline beyond:
  (4) $$f_{\mathrm{artillery}}\left(d\right)=\left\{\begin{array}{cc} \exp\left(-\frac{{\left(d-l_{\max}\right)}^{2}}{2\sigma_{\mathrm{artillery}}^{2}}\right), & d\leq l_{\max}\\ \exp\left(-\frac{d-l_{\max}}{\sigma_{\mathrm{artillery}}}\right), & d>l_{\max} \end{array}\right.$$

where $l_{\mathrm{optimal}}$ is the tank’s optimal threat range, $l_{\max}$ is the artillery vehicle’s maximum range, and $\sigma_{i}$ is the threat diffusion coefficient.

The mathematical formulations selected closely mirror tactical realities. The Gaussian distribution for tanks (Equation (2)) proves that threat effectiveness is maximized at an optimal engagement distance, penalizing both excessively close quarters (due to limited turret traverse speeds) and extreme distances (due to ballistic drop). Conversely, the exponential decay for armored vehicles (Equation (3)) mathematically models their reliance on close-range rapid deployment, where tactical advantage diminishes sharply as distance increases.

**Figure 2:**
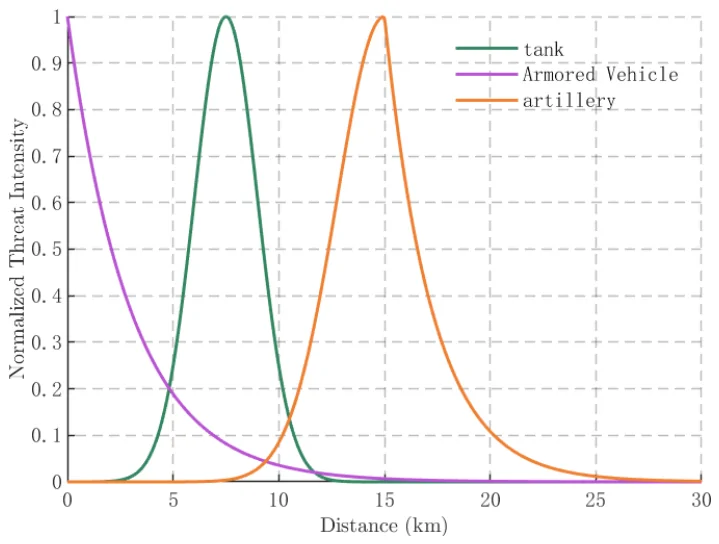
Distance-dependent distribution of threat fields for three vehicle types.

Figure 2 depicts distance-dependent threat fields: tanks peak near optimal range with gradual decay, armored vehicles decay exponentially, and artillery peaks at long range with rapid drop-off.

**Figure 3:**
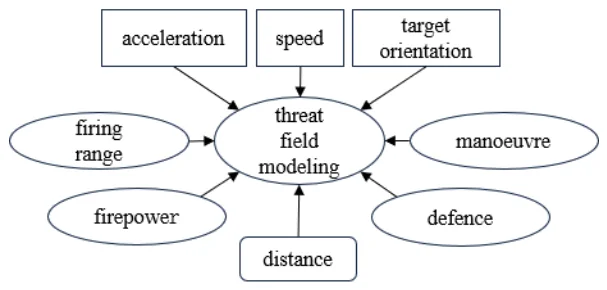
Key element diagram of the threat field.

The threat field of targets evolves dynamically. Real-time motion parameters are integrated with static attributes to model spatiotemporal threat evolution, with distance as a key coupling factor, as shown in Figure 3, enhancing situational awareness and decision-making.

**Figure 4:**
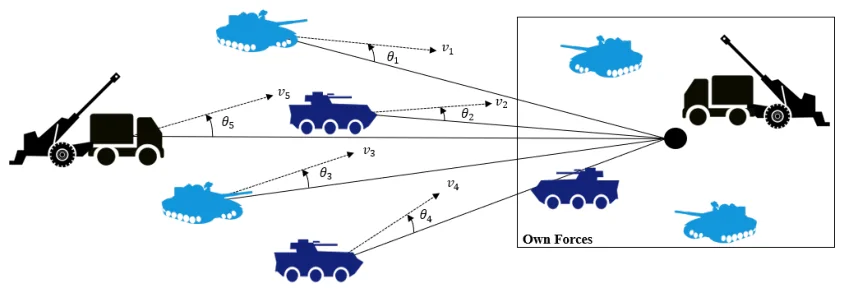
Threat field modeling diagram.

Figure 4 illustrates threat field modeling, where θ is the angle between the target’s velocity and the line to the friendly center ($0^{\circ }$–$180^{\circ }$). Key dynamic factors—velocity, acceleration, orientation, and distance—are normalized and integrated with directionality to capture the evolving threat field.

Target velocity and acceleration are critical for assessing potential threats, with their projection components toward the friendly camp defined as $v_{\|}\left(t\right)=\left|v\left(t\right)\right|\cdot \cos\left(\theta \right)$ and $a_{\|}\left(t\right)=\left|a\left(t\right)\right|\cdot \cos\left(\theta_{a}\right)$, where θ and $\theta_{a}$ denote the angles between the velocity and acceleration directions, respectively, and the line connecting to the friendly camp’s center. The normalization functions for dynamic threat factors are presented in Table 1.

$v_{0}$ is the reference velocity, typically set to 0, and $b_{v}$ controls the sensitivity of velocity to threat level; $a_{0}$ is the reference acceleration, with $b_{a}$ reflecting the influence of acceleration changes on threat level; $R_{0}$ denotes the reference distance, representing either the target’s maximum range or the distance at which its threat significantly increases, while $b_{R}$ governs the attenuation rate of threat with distance.

Multi-target threat modeling must account for both spatial distribution and temporal evolution. A temporal decay factor $\alpha \left(t\right)=e^{-\lambda_{i}\left(t-t_{0}\right)}$ is introduced, where $\lambda_{i}$ is a target-type-adaptive decay rate. To reflect tactical persistence, artillery is assigned a low decay rate ($\lambda_{\mathrm{artillery}}=0.02$) due to its sustained long-range threat, whereas armored vehicles use a higher decay rate ($\lambda_{\mathrm{armor}}=0.1$), reflecting their short-duration, highly mobile threat profiles.

**Figure 5:**
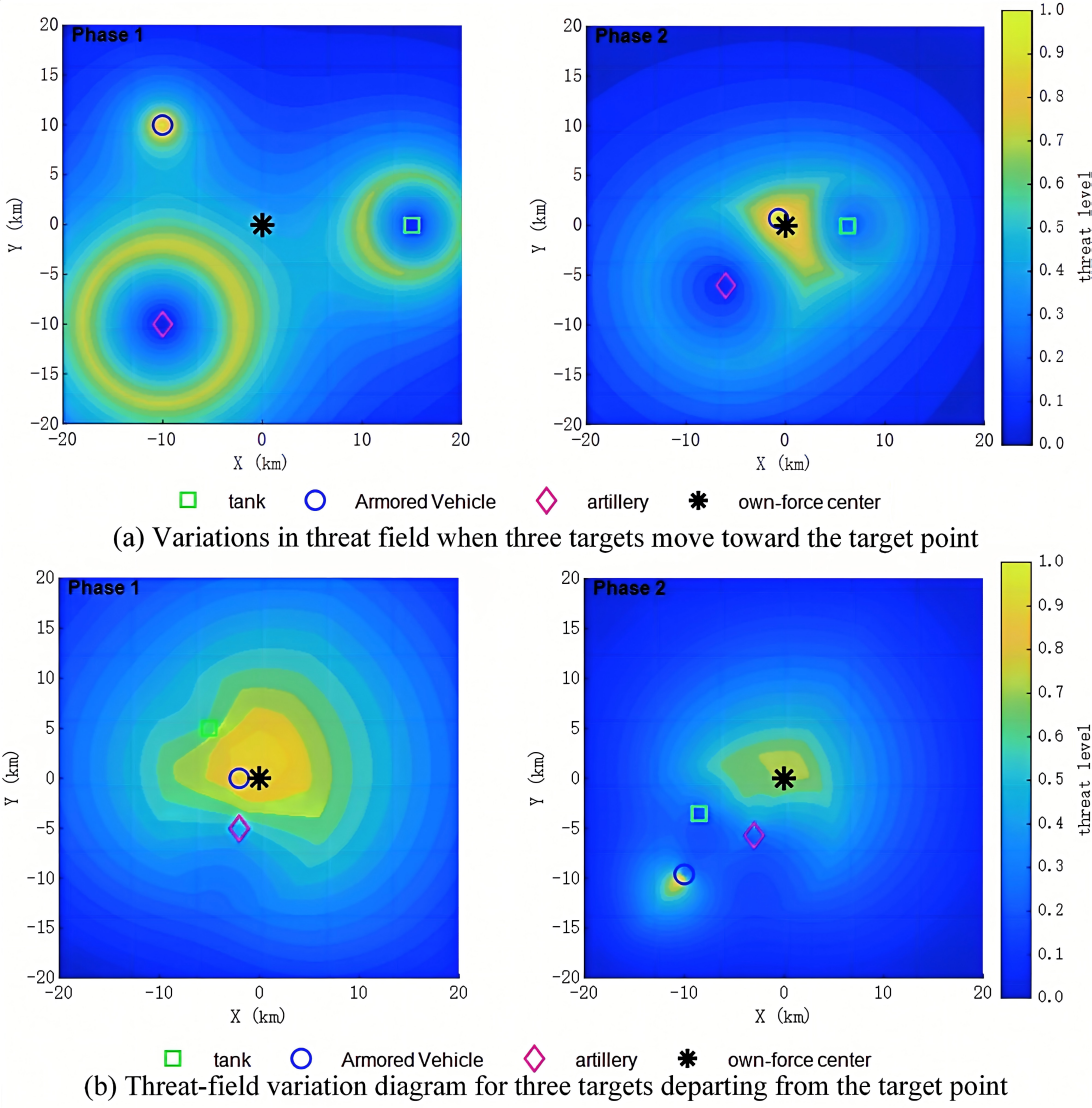
Multi-target threat landscape evolution diagram.

Based on the above dynamic threat factors, a dynamic threat field integrating spatial, temporal, and target-specific characteristics is formulated as

(5) $$W_{i}^{d}\left(x,y,t\right)=W_{i}^{s}\left(x,y\right)\cdot C_{v}\cdot C_{a}\cdot C_{R}\cdot C_{\theta }\cdot \alpha \left(t\right)$$

where $W_{i}^{s}\left(x,y\right)$ denotes the static threat intensity of target *i* at position (x,y) at time *t*. Figure 5a,b illustrate the evolution of the threat fields for the three target types under withdrawal and offensive intentions, respectively.

### 3.3. Incomplete Data Repair Mechanism

In complex environments, information gaps caused by target camouflage and environmental interference present common challenges for intent prediction. To address incomplete information, this study employs particle filtering (PF) for target state estimation. Each target’s state is characterized by latitude and longitude (lat,lon), velocity *v*, acceleration *a*, and heading θ, collectively represented as a state vector $x_{t}=\left[\mathrm{lat},\mathrm{lon},v,a,\theta \right]$.

At the initial time, *N* particles represent *N* possible target states. The state of particle *i* is denoted as $x_{t}^{\left(i\right)}={\left[\mathrm{lat},\mathrm{lon},v,a,\theta \right]}^{\left(i\right)}$, and particles are initialized based on historical trajectory data and preliminary parameter estimates.

At each time step, particles predict the target’s next state according to a motion model. The target’s motion is described using a constant acceleration model, as given in Equation (6), where Δt is the time interval and $\Delta \theta_{t}$ represents the change in heading:

(6) $$\left\{\begin{array}{cc} {\mathrm{lat}}_{t+1} & ={\mathrm{lat}}_{t}+v_{t}\cdot \Delta t+\frac{1}{2}a_{t}{\left(\Delta t\right)}^{2},\\ {\mathrm{lon}}_{t+1} & ={\mathrm{lon}}_{t}+v_{t}\cdot \Delta t+\frac{1}{2}a_{t}{\left(\Delta t\right)}^{2},\\ v_{t+1} & =v_{t}+a_{t}\cdot \Delta t,\\ \theta_{t+1} & =\theta_{t}+\Delta \theta_{t}. \end{array}\right.$$

At each time step, particle weights are updated based on current observations. Particles whose predicted states closely match the observed values receive higher weights, while those further away receive lower weights. Let the target observation be $z_{t}=\left[{\mathrm{lat}}_{\mathrm{obs}},{\mathrm{lon}}_{\mathrm{obs}},v_{\mathrm{obs}},a_{\mathrm{obs}},\theta_{\mathrm{obs}}\right]$. The particle filter computes the likelihood of each particle relative to the observation using a Gaussian distribution to update particle weights.

To prevent particle degeneration, resampling is performed after each update. This process selects particles with higher weights and generates a new particle set, maintaining particle diversity.

In multi-target scenarios, the approach is extended to cooperative particle filtering by introducing a neighborhood target angle constraint. Specifically, the motion direction of target *i* is influenced by the headings of nearby targets, allowing interactions among targets to be captured and improving the robustness of state estimation in densely populated or coordinated environments:

(7) $$\Delta \theta_{t}^{\left(i\right)}=\Delta \theta_{t}^{\left(i\right)}+\gamma \cdot \frac{1}{N-1}\sum_{j\neq i}I\left(d_{ij}<R_{\mathrm{th}}\right)\cdot \left(\theta^{\left(j\right)}-\theta^{\left(i\right)}\right)$$

where $d_{ij}$ is the Euclidean distance between targets *i* and *j*, $R_{\mathrm{th}}$ is the neighborhood threshold for mutual influence, γ quantifies motion convergence strength, and $\Delta \theta_{t}^{\left(i\right)}$ is the angular correction for target *i* at time *t*.

To handle missing observation data, the particle weight of target *i* is refined using the state information of its neighboring targets:

(8) $$w_{t}^{\left(i\right)}\propto \sum_{j\in \mathrm{Neighbor}(i)}\exp\left(-\frac{∥x_{t}^{\left(i\right)}-x_{t}^{\left(j\right)}{∥}^{2}}{2\sigma^{2}}\right)$$

where $\mathrm{Neighbor}\left(i\right)=\left\{j|d_{ij}<R_{\mathrm{th}}\right\}$ is the set of neighboring targets for target *i*, and σ is the noise parameter.

To improve the robustness of cooperative particle filtering (CPF) in complex scenarios, an adaptive cooperation coefficient γ is proposed. The coefficient γ is dynamically adjusted based on target density $\rho =N/(\pi R_{\mathrm{th}}^{2})$ and the behavioral consistency of neighboring targets, defined as

(9) $$\gamma =\gamma_{0}\cdot \min\left(1,\frac{\rho }{\rho_{\mathrm{ref}}}\right)\cdot \exp\left(-\frac{\sum_{j\in \mathrm{Neighbor}(i)}{∥\theta^{\left(j\right)}-\theta^{\left(i\right)}∥}^{2}}{\left|\mathrm{Neighbor}(i)\right|}\right)$$

where $\gamma_{0}=0.5$ is the baseline cooperation coefficient and $\rho_{\mathrm{ref}}=0.01\;{\mathrm{objects}/m}^{2}$ is the reference density, reflecting behavioral consistency via angular differences. The adaptive γ minimizes influence in sparse or heterogeneous scenarios to avoid ineffective constraints, while boosting angular correction in dense, coordinated cases, enhancing prediction accuracy for TF-GRU input.

Particle filtering updates particle weights with observation data to refine state estimates but is susceptible to particle degeneration during prolonged data gaps. To address this, this section employs DTW to quantify similarity between the current target trajectory $T^{\left(i\right)}$ and historical trajectories $T^{\left(k\right)}$, identifying the best-matching trajectory pattern to predict future motion trends.

The minimum alignment cost between the current trajectory $T^{\left(i\right)}$ and historical trajectory $T^{\left(k\right)}$ is calculated using

(10) $$D_{\mathrm{DTW}}\left(T^{\left(i\right)},T^{\left(k\right)}\right)=\min_{\pi }\sum_{(p,q)\in \pi }{∥T_{p}^{\left(i\right)}-T_{q}^{\left(k\right)}∥}^{2}$$

where π represents the alignment path, allowing nonlinear time axis scaling.

The similarity between the current trajectory $T^{\left(i\right)}$ and each historical trajectory $T^{\left(k\right)}$ in the database is computed as

(11) $$\mathrm{Sim}\left(i,k\right)=\exp\left(-\frac{D_{\mathrm{DTW}}\left(T^{\left(i\right)},T^{\left(k\right)}\right)}{\beta }\right)$$

where β is a tuning parameter. The top *K* historical trajectories with the highest similarity are selected to enhance prediction robustness.

Using the threat field data from the selected trajectories, the dynamic threat field increment for the missing target is predicted as

(12) $$\Delta W_{\mathrm{predicted}}^{\left(i\right)}=\sum_{k=1}^{K}\mathrm{Sim}\left(i,k\right)\cdot W_{d}^{\left(k\right)}\left(t\right)$$

The dynamic threat field of the missing target is then updated based on the predicted increment:

(13) $$W_{d}^{\left(i\right)}\left(t+1\right)=W_{d}^{\left(i\right)}\left(t\right)\cdot \alpha \left(t\right)+\Delta W_{\mathrm{predicted}}^{\left(i\right)}$$

In this framework, DTW is employed to retrieve the most similar historical trajectories, from which the corresponding state information $x_{\mathrm{dtw}}\left(t\right)$ is extracted. This state is reformatted to match the particle filter output $x_{\mathrm{pf}}\left(t\right)$, after which a weighted fusion of the two estimates is performed.

To ensure high-accuracy state estimation under both short- and long-term observation gaps, a dynamic repair mechanism integrating particle filtering (PF) with dynamic time warping (DTW) is proposed. The method dynamically adjusts the weights of PF and DTW according to the duration of missing observations $t_{\mathrm{gap}}$, thereby exploiting PF’s real-time tracking capability for short gaps and DTW’s long-sequence matching ability for extended gaps.

Let $t_{\mathrm{threshold}}$ denote the switching threshold for weight allocation. The PF weight is $w_{\mathrm{pf}}$, and the DTW weight is $w_{\mathrm{dtw}}$, constrained by $w_{\mathrm{pf}}+w_{\mathrm{dtw}}=1$. The weights are adjusted dynamically according to $t_{\mathrm{gap}}$: short-term loss ($t_{\mathrm{gap}}<t_{\mathrm{threshold}}$) relies on PF, while long-term loss ($t_{\mathrm{gap}}\geq t_{\mathrm{threshold}}$) relies on DTW:

(14) $$w_{\mathrm{dtw}}=\min\left(\frac{t_{\mathrm{gap}}}{t_{\mathrm{threshold}}},1\right),\;w_{\mathrm{pf}}=1-w_{\mathrm{dtw}}$$

PF and DTW provide state estimates $x_{\mathrm{pf}}\left(t\right)$ and $x_{\mathrm{dtw}}\left(t\right)$, respectively. The final state estimate is obtained by weighted fusion of the two results:

(15) $$x_{\mathrm{final}}\left(t\right)=w_{\mathrm{pf}}\cdot x_{\mathrm{pf}}\left(t\right)+w_{\mathrm{dtw}}\cdot x_{\mathrm{dtw}}\left(t\right)$$

Finally, the predicted state is used to update the dynamic threat field to ensure its validity under data loss conditions:

(16) $$W_{d}^{\left(i\right)}\left(t+1\right)=W_{d}^{\left(i\right)}\left(t\right)\cdot \alpha \left(t\right)+\Delta W_{\mathrm{predicted}}^{\left(i\right)}+\eta \cdot \frac{1}{N_{\mathrm{neighbor}}}\sum_{j\in \mathrm{Neighbor}(i)}W_{d}^{\left(j\right)}\left(t\right)$$

where α(t) is the time decay factor, η∈[0,1] is the neighborhood threat cooperation coefficient, and $N_{\mathrm{neighbor}}$ is the number of neighboring targets.

**Figure 6:**
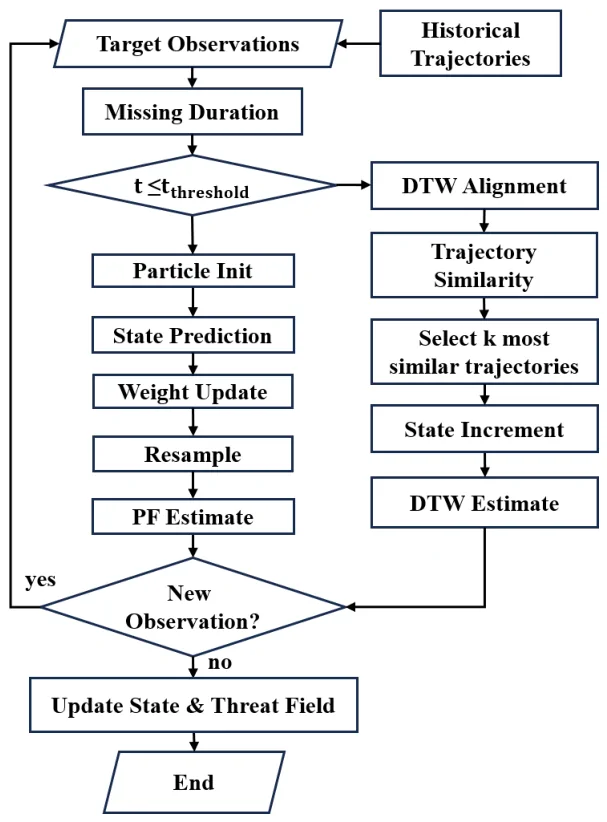
Flowchart of the PF–DTW dynamic repair mechanism.

The PF-DTW dynamic repair mechanism is illustrated in Figure 6. This mechanism simultaneously supplements trajectory data and the dynamic threat field, thereby ensuring the completeness of TF–GRU inputs. The final state estimate $x_{\mathrm{final}}\left(t\right)$ is directly mapped to the trajectory data.

Compared to single PF (prone to degeneration in long-term gaps), single DTW (lacking short-term responsiveness), and linear interpolation [28] (relying solely on geometry), the PF-DTW method integrates real-time estimation with historical matching, significantly enhancing trajectory and threat field robustness and accuracy.

### 3.4. TF-GRU Network for Intent Prediction

**Figure 7:**
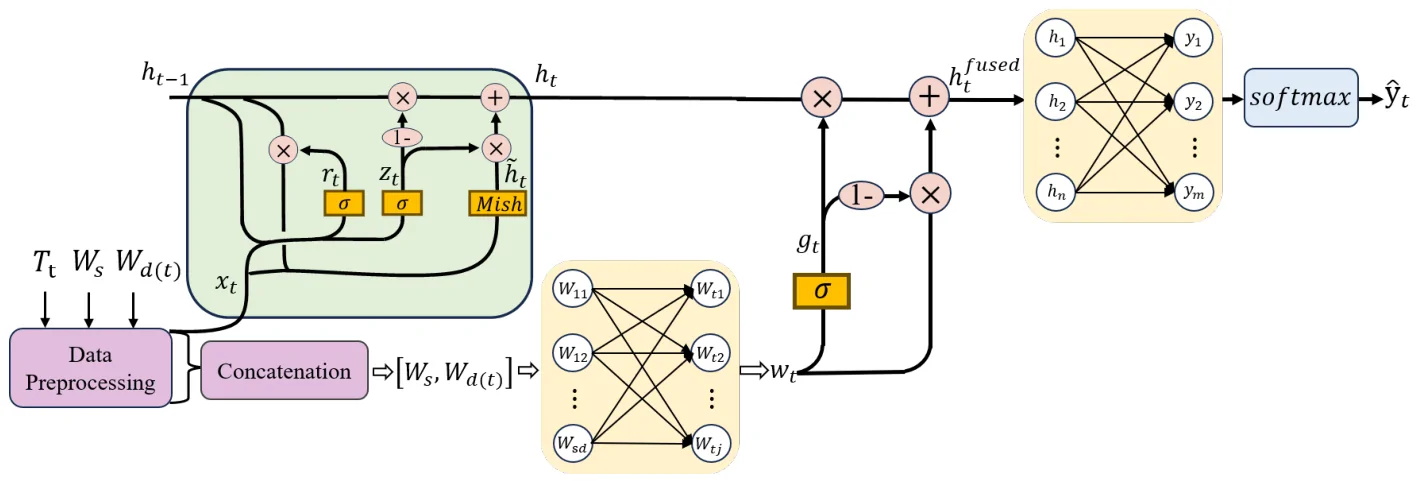
Framework of the single-target GRU model based on target trajectory and threat field.

This study proposes a GRU-based TF-GRU model to predict ground multi-target intent by integrating target trajectories and threat fields, capturing dynamic behavioral patterns over time. The model fuses trajectory data $T_{t}=\left[{\mathrm{lat}}_{t},{\mathrm{lon}}_{t},v_{t},a_{t},\theta_{t}\right]$ with static ($W_{s}$) and dynamic ($W_{d}\left(t\right)$) threat fields, enhancing intent inference accuracy. Under missing data, the PF-DTW mechanism reconstructs $T_{t}$ by combining particle filtering for state estimation with dynamic time warping for temporal alignment, ensuring continuity in reconstructed sequences. The normalized input vector $x_{t}=\left[T_{t},W_{s},W_{d}\left(t\right)\right]$ forms the sequence $\left\{x_{1},\ldots ,x_{T}\right\}$, which is fed into an enhanced GRU framework (Figure 7).

As an RNN variant, GRU mitigates gradient vanishing with gating mechanisms [29]. The proposed model incorporates three key enhancements:

1. Adaptive Mish Activation: The Mish function, Mish(x)=x·tanh(Softplus(x)) with $\mathrm{Softplus}\left(x\right)=\ln\left(1+e^{x}\right)$, preserves non-zero gradients for nonlinear feature capture [30]. The candidate state updates as ${\tilde{h}}_{t}=\mathrm{Mish}\left(W_{h}\left[r_{t}⊙h_{t-1},x_{t}\right]+b_{h}\right)$.
2. Adaptive Xavier Initialization: Gates ($W_{r}$, $W_{z}$) use orthogonal initialization, while $W_{h}∼N\left(0,1/n_{\mathrm{in}}\right)$ is scaled by $\delta =1/\max(\sigma_{\mathrm{traj}}^{2},\sigma_{\mathrm{threat}}^{2})$, ensuring stable training [31].
3. Threat Field Adaptive Gating: A fully connected layer maps $\left[W_{s},W_{d}\left(t\right)\right]$ to $w_{t}=\mathrm{ReLU}\left(W_{\mathrm{threat}}\left[W_{s},W_{d}\left(t\right)\right]+b_{\mathrm{threat}}\right)$. Gating vector $g_{t}=\sigma \left(W_{g}w_{t}+b_{g}\right)$ fuses $h_{t}$ and $w_{t}$ into $h_{t}^{\mathrm{fused}}=g_{t}⊙h_{t}+\left(1-g_{t}\right)⊙w_{t}$, dynamically balancing threat and trajectory data.

The improved GRU updates are governed by

(17) $$\left\{\begin{array}{cc} r_{t} & =\sigma (W_{r}\left[h_{t-1},x_{t}\right]+b_{r})\\ z_{t} & =\sigma (W_{z}\left[h_{t-1},x_{t}\right]+b_{z})\\ {\tilde{h}}_{t} & =\mathrm{Mish}(W_{h}\left[r_{t}⊙h_{t-1},x_{t}\right]+b_{h})\\ h_{t} & =\left(1-z_{t}\right)⊙h_{t-1}+z_{t}⊙{\tilde{h}}_{t}\\ h_{t}^{\mathrm{fused}} & =g_{t}⊙h_{t}+\left(1-g_{t}\right)⊙w_{t} \end{array}\right.$$

Intent prediction outputs the probability distribution via

(18) $${\hat{y}}_{t}=\mathrm{softmax}\left(W_{o}h_{t}^{\mathrm{fused}}+b_{o}\right)$$

while multi-target prediction uses

(19) $${\hat{y}}_{t}^{\left(i\right)}=\mathrm{softmax}\left(W_{o}^{\left(i\right)}h_{t}^{(i),\mathrm{fused}}+b_{o}^{\left(i\right)}\right)$$

fused globally with weight

(20) $$\omega^{\left(i\right)}=\tau W_{d}^{\left(i\right)}\left(t\right)+\left(1-\tau \right)W_{s}^{\left(i\right)}$$

where τ∈[0,1] balances threat factors. The final intent is

(21) $${\hat{Y}}_{t}\left(c\right)=\frac{1}{\sum_{i=1}^{n}\omega^{\left(i\right)}}\sum_{i=1}^{n}\omega^{\left(i\right)}\cdot {\hat{y}}_{t,c}^{\left(i\right)}$$

selecting the highest-probability category.

In these equations, the reset gate $r_{t}$ determines how much of the past trajectory and threat information should be forgotten, which is particularly crucial when sudden tactical maneuvers occur. The update gate $z_{t}$ balances the integration of the new candidate state ${\tilde{h}}_{t}$ with the previous hidden state $h_{t-1}$. The use of the Mish activation function in the candidate state formulation prevents gradient vanishing during the learning of complex, nonlinear multi-target interactions. Finally, the threat-adaptive gating vector $g_{t}$ acts as a dynamic weighting mechanism, smoothly prioritizing between pure kinematic trajectory features and semantic threat field features based on the current battlefield context.

### 3.5. Training Process and Loss Function

The TF-GRU model is trained to predict multi-target behavior and intent. The dataset contains 40 trajectories representing tactical scenarios involving five coordinated targets. Data are split into training, validation, and test sets in a 7:1.5:1.5 ratio, each recording position, velocity, acceleration, and heading. Intent labels are expert-annotated into five categories (Table 2).

The model was implemented in Python 3.9 using PyTorch and trained on an NVIDIA RTX 4060 GPU. Model parameters were initialized using adaptive Xavier initialization and optimized for 200 epochs with mini-batch SGD (batch size 32; initial learning rate 0.001) and cosine annealing. The checkpoint with the best Macro-F1 score was retained. Incomplete data were randomly masked and repaired using PF-DTW, after which the repaired trajectory data were fused with threat field features through adaptive gating.

The loss function combines (i) single-target cross-entropy, (ii) a threat-weighted global intent loss to integrate predictions across targets, and (iii) an entropy-based regularization term that suppresses overconfident predictions under missing data. The overall objective is

(22) $$L=L_{\mathrm{global}}+\alpha L_{\mathrm{reg}},\;\alpha =0.1$$

where $L_{\mathrm{global}}$ denotes the global threat-weighted classification loss and $L_{\mathrm{reg}}$ the entropy regularization term. This formulation balances classification accuracy and robustness in incomplete-information scenarios.

To justify the empirical selection of the regularization weight α, parameter-tuning experiments were conducted under the missing-data scenario. As shown in Table 3, varying α significantly impacts the model’s predictive uncertainty and accuracy. A minimal weight (α=0.01) fails to suppress overconfident misclassifications, while an excessive weight (α=0.5) severely penalizes the network’s confidence, hindering effective convergence. The selected weight of α=0.1 optimally balances the suppression of overconfidence with the preservation of accurate classification capabilities.

Pseudocode is provided to outline the TF-GRU workflow, highlighting threat field construction, PF-DTW-based trajectory repair, and cooperative particle filtering for multi-target interaction, offering a clear, language-independent overview of TF-GRU (Algorithm 1).
**Algorithm 1** TF-GRU training and loss function**Require:** Dataset *D*, Labels *Y***Ensure:** Best parameters $\theta^{*}$, Best F1  1:**Init:** $\mathrm{bs}\leftarrow 32,\mathrm{eps}\leftarrow 200,\eta \leftarrow 10^{-3},\lambda \leftarrow 0.1$  2:$\left(D_{tr},D_{val},D_{te}\right)\leftarrow$Split(D,[7,1.5,1.5])  3:m←TFGRU,o←SGD(m,η),s←Cos(o)  4:**for** ep=1 **to** eps **do**  5:      **for** *b* **in** GetBatches$\left(D_{tr},D_{val},D_{te}\right)$ **do**  6:            tr,Y←b.traj,b.y;S,D← Static (tr),Dyn(*tr*)  7:            **if** Missing(b) **then** tr←PF-DTW(tr)  8:            **end if**  9:            X← Norm (CAT(tr,S,D));h←m. InitH (bs)10:            **for** t=1 **to** *T* **do**11:                  $g_{t}\leftarrow \sigma \left(\mathrm{FC}\left(\left[S_{t},D_{t}\right]\right)\right);\;X_{t}\leftarrow X_{t}⊙g_{t}$12:                  h← ImpGRU $\left(X_{t},h\right)$13:            **end for**14:            ${\hat{y}}_{gl}\leftarrow \sum$ Weight (S,D)· Softmax (FC(h))15:            $L\leftarrow -\frac{1}{n}\sum Y\log\left({\hat{y}}_{gl}\right)+\frac{\lambda }{n}\sum {\hat{y}}_{gl}\log\left({\hat{y}}_{gl}\right)$16:            
*o*.Zero(); L.Back(); *o*.Step()
17:      **end for**18:      *s*.Step();f1← Eval $\left(m,D_{val}\right)$19:      **if** f1>best **then**20:            $\mathrm{best}\leftarrow f1,\theta^{*}\leftarrow m.$State()21:      **end if**22:**end for**23:Save($\theta^{*}$, ’best_model.pt’)

## 4. Experimental Setup and Results Analysis

### 4.1. Dataset Introduction

The characteristics and number of experimental targets are detailed in Table 4. Firepower, defense, and mobility are normalized to the [0,1] interval using the expert scoring method [32], with the threat diffusion coefficient set based on tactical requirements. Importance weights are derived via the Analytic Hierarchy Process (AHP) [33], involving pairwise comparisons of firepower, defense, and mobility to construct a consistent matrix, yielding normalized weights $\omega_{i}$ such that $\sum \omega_{i}=1$. The threat diffusion coefficient follows a Gaussian model, with $\sigma_{i}=k\cdot l_{\mathrm{optimal}}$ (k=0.1–0.2), ensuring alignment with tactical significance and operational needs.

The parameter settings in Table 4, including firepower, defense, and mobility coefficients, are derived from standard Computer Generated Forces (CGF) military simulation databases. The optimal range and threat diffusion coefficients were established through expert domain knowledge, reflecting the physical constraints of respective weapon systems in real-world ground engagements.

**Figure 8:**
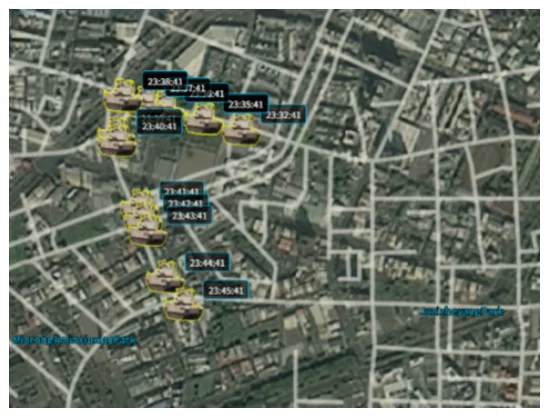
An example of the simulation results.

The simulation experiment utilizes real road network data from a specific region, loaded from OpenStreetMap using OSMnx (roads.graphml). The largest strongly connected subgraph is extracted as the simulation foundation to ensure target movement paths adhere to actual road constraints, replicating realistic motion logic. Each trajectory represents a complete tactical scenario involving coordinated or independent actions of five targets. Target motion states evolve over time, with tactical intentions annotated by human experts. Each trajectory spans 3600 time steps (1 s per step), with a total of 40 trajectories. The dataset is split into training, validation, and test sets in a 7:1.5:1.5 ratio to ensure comprehensive model training and evaluation. An example of the simulation results is shown in Figure 8.

The visualization highlights the OSMnx road network overlay. The yellow nodes represent the coordinate sequence of target movements, while the blue annotations indicate the dynamic intent classification output at specific waypoints, demonstrating the model’s continuous tracking capability in a constrained urban terrain.

This study integrates the complex environment of multi-target coordinated operations, target motion characteristics, and tactical mission context to define an output space encompassing five intent types. Raw trajectory data undergoes spatial feature extraction and threat field computation to generate model inputs. First, the spherical distance between enemy targets and the friendly center is calculated using the haversine formula [34]:

(23) $$d=2R\arcsin\left(\sqrt{\sin^{2}\frac{\Delta \mathrm{lat}}{2}+\cos\left({\mathrm{lat}}_{1}\right)\cos\left({\mathrm{lat}}_{2}\right)\sin^{2}\frac{\Delta \mathrm{lon}}{2}}\right)$$

where R=6371km is the Earth’s radius, and Δlat and Δlon are the latitude and longitude differences in radians.

Next, the azimuthal deviation between the target’s velocity direction and the friendly enemy line is computed: the friendly enemy line direction $\theta_{\mathrm{link}}$ is determined via spherical trigonometry, the velocity direction $\theta_{\mathrm{vel}}$ is derived from target orientation data, and the final angle is given by $\theta =\min\left(|\theta_{\mathrm{vel}}-\theta_{\mathrm{link}}|,\;360^{\circ }-\left|\theta_{\mathrm{vel}}-\theta_{\mathrm{link}}\right|\right)$.

Multi-target threat values are aggregated and normalized to the [0,1] interval. All numerical features are normalized per Table 1 formulas, and time series data are segmented into input samples using a 30 s window.

### 4.2. Experimental Setup

#### 4.2.1. Hyperparameter Settings

The experiments were conducted on an NVIDIA GeForce RTX 4060 GPU with CUDA 11.7 for accelerated computing, using NumPy (version: 1.26.4) and PyTorch (version: 2.2.2) in a Python 3.9 environment for data generation, threat field modeling, and network training. The TF-GRU model’s hyperparameters are set as follows: adaptive Xavier weight initialization dynamically adjusts weight scales based on input channel variance. The optimizer employs mini-batch Stochastic Gradient Descent (SGD) with a batch size of 32, an initial learning rate of 0.001, and a cosine annealing learning rate schedule (minimum learning rate $10^{-5}$, cycle of 10 epochs). Training spans 200 epochs, with Macro-F1 score evaluation on the validation set every 5 epochs, saving the optimal model.

**Figure 9:**
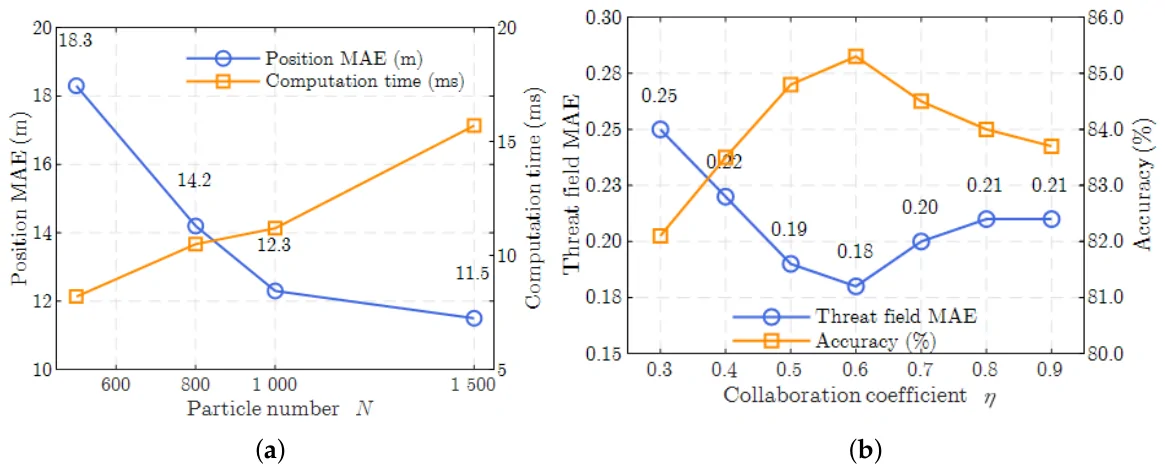
Selection of particle number and collaboration coefficient. (**a**) Selection of the number of particles. (**b**) Selection of the correlation coefficient.

For the PF-DTW mechanism, particle filtering uses N=1000 particles with process noise variance σ=0.01; DTW has an adjustment factor β=50 and a historical trajectory library capacity of 500 entries; the dynamic switching threshold is $t_{\mathrm{threshold}}=15\;s$. Cooperative particle filtering sets the neighborhood threshold at the tactical distance (500 m), with a cooperation coefficient η=0.6, dynamic threat proportion coefficient τ=0.5, and regularization weight α=0.1. The selection of parameters is shown in Figure 9.

#### 4.2.2. Evaluation Metrics and Baseline Models

The performance of the proposed TF-GRU model is evaluated using three key metrics: accuracy, Macro-F1, and uncertainty. Accuracy reflects the proportion of correctly predicted samples, serving as an overall measure of classification performance, while Macro-F1 represents the average F1 score across all intent categories to ensure balanced multi-class prediction. The mean entropy of the predicted probability distribution is used to quantify prediction uncertainty, calculated as

(24) $$\mathrm{Uncertainty}=\frac{1}{N}\sum_{i=1}^{N}\sum_{c=1}^{C}{\hat{y}}_{i,c}\log\left({\hat{y}}_{i,c}\right)$$

where *N* is the number of samples, *C* is the number of classes, and ${\hat{y}}_{i,c}$ is the predicted probability for class *c* of sample *i*. Lower values indicate higher confidence in the model’s predictions.

To validate TF-GRU’s effectiveness against the latest advancements in intent prediction, four representative state-of-the-art baseline models are compared. Transformer [17] utilizes attention mechanisms to capture global, long-range temporal dependencies, representing the latest breakthrough in sequence modeling but remaining less effective with incomplete data. BiLSTM-TCN [9] integrates Bidirectional Long Short-Term Memory networks and Temporal Convolutional Networks as a competitive architecture specifically designed for enhanced intent recognition, yet struggles with multi-target coordination. Additionally, CNN-LSTM [35] combines convolutional neural networks for spatial feature extraction and LSTMs for temporal modeling, which is effective for spatio-temporal prediction but less robust under missing data. DBN [14] employs adversarial intent prediction and Intelligence, Surveillance, and Reconnaissance (ISR) reliability submodels for interpretable uncertainty quantification, but relies heavily on prior data and adapts poorly in data-scarce scenarios. All models use the same dataset, hyperparameters, optimizers, and learning rate schedules as TF-GRU for a fair comparison.

### 4.3. Experimental Results

#### 4.3.1. Results of the Proposed Model

**Figure 10:**
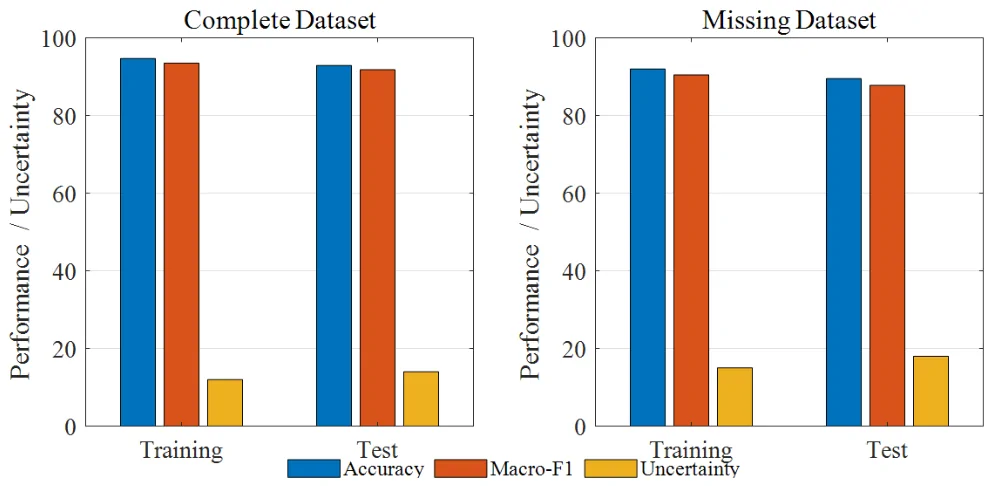
Comparison of model performance under complete and missing data scenarios.

This study comprehensively evaluated the TF-GRU model for trajectory intent prediction using the OSMnx road network dataset, comprising 40 trajectories, 5 targets, 3600 time steps, and 5 intent categories. Experiments included both complete data and incomplete information scenarios to assess model performance under varying conditions. Performance metrics—accuracy, Macro-F1 score, and prediction uncertainty—are reported in Table 5 and Table 6 and Figure 10.

In the complete data scenario, TF-GRU exhibited superior fitting and generalization capabilities. Table 5 indicates an accuracy of 94.7%, a Macro-F1 score of 93.5%, and an uncertainty of 0.12 on the training set; on the test set, accuracy was 92.9%, Macro-F1 score was 91.8%, and uncertainty rose slightly to 0.14, reflecting stable predictions on unseen data.

In incomplete information scenarios, the model demonstrated robust performance, achieving a training set accuracy of 92.0%, a Macro-F1 score of 90.5%, and an uncertainty of 0.15; on the test set, accuracy was 89.5%, Macro-F1 score was 87.8%, and uncertainty was 0.18. Table 6 shows that per-intent accuracy exceeded 85% in incomplete data scenarios. Figure 10 uses bar charts to compare performance across scenarios, confirming TF-GRU’s high accuracy and stability despite data missingness.

Overall, TF-GRU excels in both complete and incomplete data scenarios, delivering high accuracy and low uncertainty, thus providing a robust solution for trajectory intent prediction.

To rigorously verify the model’s robustness, we conducted supplementary experiments varying the missing data ratio from 10% to 40%, as summarized in Table 7. A two-tailed *t*-test confirmed that the performance degradation is statistically significantly lower (p<0.05) compared to baseline models at severe missing ratios.

#### 4.3.2. Comparison with Other Models

**Figure 11:**
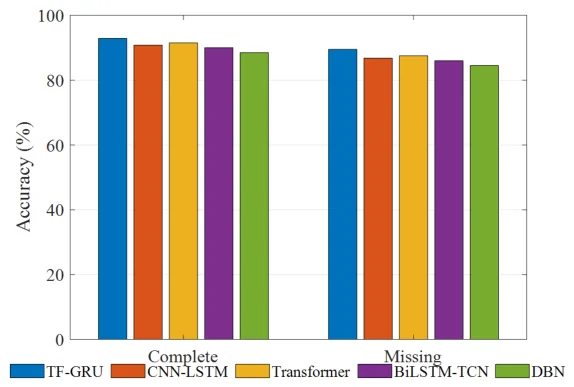
Comparison of accuracy across different models.

**Figure 12:**
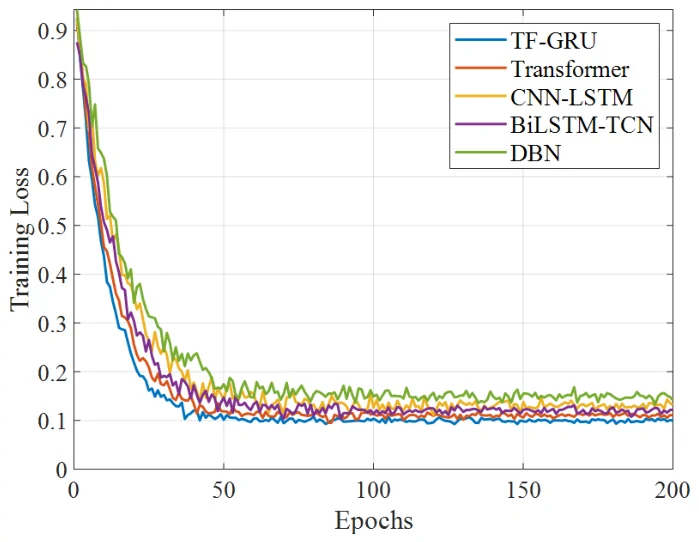
Comparison of model loss curves.

To evaluate the superiority of the TF-GRU model, it was compared with four representative models-CNN-LSTM, Transformer, BiLSTM-TCN, and DBN-using the OSMnx road network dataset. The experiments covered both complete and incomplete data scenarios, with accuracy as the performance metric, as shown in Table 8 and Figure 11. The training process consisted of 200 epochs, with loss convergence illustrated in Figure 12.

Figure 11 presents the test set accuracy comparison of the five models. On the complete dataset, TF-GRU achieved the highest accuracy of 92.9%, outperforming CNN-LSTM (90.8%), Transformer (91.5%), BiLSTM-TCN (90.0%), and DBN (88.5%). In incomplete data scenarios, TF-GRU maintained robustness with an accuracy of 89.5%, higher than CNN-LSTM (86.8%), Transformer (87.5%), BiLSTM-TCN (86.0%), and DBN (84.5%). These results demonstrate that TF-GRU provides superior prediction accuracy and maintains stability under missing data conditions.

Figure 12 shows the training loss convergence curves on the complete dataset. TF-GRU converged the fastest, with loss stabilizing at approximately 0.10, outperforming the other models. All models’ loss curves exhibited a clear exponential decay, but TF-GRU’s rapid decline and low stable loss reflect the efficiency of its optimized gated recurrent structure in learning trajectory intent patterns.

In summary, TF-GRU surpasses CNN-LSTM, Transformer, BiLSTM-TCN, and DBN in both accuracy and convergence speed, particularly under incomplete data conditions, providing an efficient and robust solution for trajectory intent prediction tasks.

#### 4.3.3. Ablation Experiment Results

To validate the contributions of the key components in the TF-GRU model, ablation experiments were conducted to evaluate the impact of the threat field, path planning dynamic time warping (PF-DTW), and the improved GRU module on trajectory intent prediction performance. The experiments used the OSMnx road network dataset. Performance metrics included accuracy, Macro-F1 score, and prediction uncertainty, as reported in Table 9.

The complete TF-GRU model was compared with three variants: without threat field, without PF-DTW, and with standard GRU. On the complete dataset, TF-GRU achieved the highest accuracy of 92.9%, slightly outperforming no PF-DTW (91.5%) and substantially surpassing GRU (85.7%) and no threat field (82.3%). In the missing data scenario, TF-GRU maintained robustness with an accuracy of 89.5%, higher than no threat field (77.8%), GRU (79.6%), and no PF-DTW (74.8%). Table 9 further shows that TF-GRU’s Macro-F1 scores (complete: 91.8%, missing: 87.8%) and uncertainty (complete: 0.14, missing: 0.18) were superior to no threat field (0.24, 0.30), no PF-DTW (0.15, 0.35), and GRU (0.20, 0.27).

The ablation results show that the threat field substantially improves global feature extraction by modeling environmental risks, with its removal causing a marked performance drop under missing data conditions. PF-DTW is essential for trajectory feature matching in incomplete scenarios, and omitting it sharply reduces accuracy and Macro-F1, highlighting its role in robustness, while its effect on complete data is minor due to compensation from the improved GRU and threat field. The improved GRU enhances sequence modeling, and replacing it with a standard GRU lowers performance, confirming its importance in capturing long-term dependencies. The full TF-GRU model, combining all components, delivers superior accuracy, Macro-F1, and uncertainty, demonstrating an optimized design.

In summary, the ablation experiments confirm the indispensable contributions of the threat field, PF-DTW, and improved GRU to the TF-GRU model. Particularly in incomplete data scenarios, the complete model exhibits strong robustness and efficiency, providing an optimized solution for trajectory intent prediction tasks.

To systematically validate the empirical configuration of the PF–DTW dynamic switching threshold ($t_{\mathrm{threshold}}$), a comprehensive sensitivity analysis was conducted across observation gap intervals ranging from 5s to 30s. The primary objective of this analysis is to identify the optimal transition point that effectively balances the short-term real-time kinematic tracking superiority of the particle filter (PF) with the long-term historical pattern-matching resilience of dynamic time warping (DTW). As quantitatively summarized in Table 10, a threshold of 15s yields the optimal predictive performance, achieving a peak accuracy of 89.5% and a Macro-F1 score of 87.8%. The experimental results reveal a clear performance degradation at both temporal extremes. When the threshold is configured too short (e.g., 5s or 10s), the mechanism prematurely abandons the kinematic accuracy of PF; during these brief gaps, PF remains highly capable of maintaining high-confidence state estimates, and an early transition to DTW introduces unnecessary historical alignment noise that lowers overall accuracy (e.g., 86.4% at 5s). Conversely, extending the threshold too far (e.g., 20s and 30s) exposes the inherent limitations of standard particle filtering. Without continuous observation updates over extended periods, the PF mechanism suffers from severe particle degeneration and uncorrected state drift before DTW is even activated, ultimately degrading the intent prediction accuracy to 84.6% at 30s. Therefore, the 15s threshold serves as a critical inflection point, ensuring that the model seamlessly transitions to historical trajectory matching precisely when real-time state estimation begins to fail.

#### 4.3.4. Discussion and Outlook

The experimental results demonstrate that TF-GRU achieves high accuracy and robustness in complex environments through threat field modeling, PF-DTW dynamic repair, and multi-target coordination mechanisms. Compared with CNN-LSTM, Transformer, BiLSTM-TCN, and DBN, TF-GRU performs particularly well in incomplete information scenarios, thanks to its dynamic data repair and explicit threat modeling capabilities. While BiLSTM-TCN shows strong single-target temporal modeling performance, it lacks multi-target coordination; DBN is constrained by prior data requirements, limiting its performance in dynamic scenarios. TF-GRU overcomes these limitations through the integration of threat field modeling and cooperative particle filtering.

Currently, the model is designed for ground targets. Future research directions include adapting the approach to aerial targets by adjusting threat field parameters, extending particle filter dimensions to three-dimensional space, and optimizing GRU temporal modeling. Other priorities involve acquiring real datasets, enhancing real-time performance (e.g., reducing PF-DTW iteration time), and introducing nonlinear motion models to improve generalization and practical applicability. Further exploration of multimodal data fusion, such as combining image and radar data, could enhance the model’s adaptability to complex environments.

Regarding computational complexity, the PF mechanism operates at O(N), where *N* is the particle count, taking approximately 12.3ms per step. The DTW historical matching operates at $O(L^{2}K)$, where *L* is the sequence length and *K* is the trajectory library size. With K=500, DTW introduces an overhead of approximately 45ms per activation. Because DTW is only triggered during extended data gaps (>15s), the combined PF–DTW mechanism maintains an average runtime of approximately 18ms per time step, well within the real-time requirements of 1Hz sensor update rates.

## 5. Conclusions

This study proposes TF-GRU, a Gated Recurrent Unit-based model integrating static and dynamic threat field modeling with a particle filtering–dynamic time warping dynamic switching strategy to enhance enemy intent prediction in complex environments. By linking target attributes to tactical intentions, PF-DTW corrects short-term data gaps in real time via particle filtering and addresses long-term gaps through DTW-based historical trajectory matching, while cooperative particle filtering boosts robustness in multi-target scenarios. Experimental results validate TF-GRU’s effectiveness in mitigating information incompleteness, with potential for real-world deployment. Future work will extend the model to aerial targets by tuning threat parameters, incorporating 3D particle filtering, and refining GRU temporal modeling, alongside introducing nonlinear motion models, real datasets, and optimized real-time performance for broader applicability.

## Figures and Tables

**Table 1:** Dynamic threat factor definitions.

Threat Factor	Description	Normalization Function
*v*	Velocity	$C_{v}=\frac{1}{1+e^{-\frac{v_{\|}\left(t\right)-v_{0}}{b_{v}}}}$
*a*	Acceleration	$C_{a}=\frac{1}{1+e^{-\frac{a_{\|}\left(t\right)-a_{0}}{b_{a}}}}$
θ	Orientation	$C_{\theta }=1-\frac{\left|\theta (t)\right|}{180^{\circ }}$
*R*	Distance	$C_{R}=e^{-{\left(\frac{R\left(t\right)-R_{0}}{b_{R}}\right)}^{2}}$

**Table 2:** Intent encoding.

Intent Type	Encoding
Attack	0
Reconnaissance	1
Withdrawal	2
Action	3
Strike	4

**Table 3:** Parameter tuning of the entropy regularization weight (α) under the missing-data scenario.

Regularization Weight (α)	Acc (%)	F1 (%)	Unc
0.01	86.2	84.5	0.08
0.05	88.1	86.4	0.12
0.10 (Selected)	89.5	87.8	0.18
0.20	87.6	86.0	0.25
0.50	84.3	82.7	0.38

**Table 4:** Target characteristics and threat parameter settings.

Target Type	Quantity	Range (m)	Firepower	Defense	Mobility	Optimal Range (m)	Importance ($\omega_{i}$)	Threat Diffusion Coefficient ($\sigma_{i}$)
Tank	2	3000	0.9	0.8	0.6	1500	0.5	300
Armored Vehicle	2	1000	0.6	0.5	0.8	500	0.2	100
Artillery	1	8000	0.7	0.4	0.4	5000	0.3	800

**Table 5:** Model performance metrics.

Dataset	Complete Dataset			Missing Dataset		
	Acc (%)	F1 (%)	Unc	Acc (%)	F1 (%)	Unc
Train	94.7	93.5	0.12	92.0	90.5	0.15
Test	92.9	91.8	0.14	89.5	87.8	0.18

**Table 6:** Mean accuracy for each intent.

Intent	Mean Accuracy (%)
Reconnaissance	89.40
Attack	93.30
Withdrawal	87.00
Action	86.25
Strike	92.83

**Table 7:** Robustness analysis under varying missing data ratios.

Missing Ratio	Acc (%)	F1 (%)	Uncertainty
10%	91.2	90.1	0.14
20% (Baseline)	89.5	87.8	0.18
30%	85.3	84.0	0.23
40%	79.1	77.5	0.31

**Table 8:** Comparison of model performance metrics.

Model	Accuracy (%)	
	Complete Dataset	Missing Dataset
TF-GRU	92.9	89.5
CNN-LSTM	90.8	86.8
Transformer	91.5	87.5
BiLSTM-TCN	90.0	86.0
DBN	88.5	84.5

**Table 9:** Ablation experiment performance comparison.

Model	Components			Complete Dataset			Missing Dataset		
	Threat Field	PF-DTW	Imp. GRU	Acc (%)	F1 (%)	Unc	Acc (%)	F1 (%)	Unc
TF-GRU (Proposed)	✔	✔	✔	**92.9**	**91.8**	**0.14**	**89.5**	**87.8**	**0.18**
w/o Threat Field	-	✔	✔	82.3	80.5	0.24	77.8	75.9	0.30
w/o PF-DTW	✔	-	✔	91.5	90.0	0.15	74.8	68.6	0.35
w/o Improved GRU	✔	✔	-	85.7	84.2	0.20	79.6	78.1	0.27

Bold values indicate the highest performance.

**Table 10:** Sensitivity analysis of PF–DTW switching threshold ($t_{\mathrm{threshold}}$).

$t_{\mathrm{threshold}}$	Acc (%)	F1 (%)
5s	86.4	85.1
10s	88.1	86.9
15s (Selected)	89.5	87.8
20s	87.2	86.0
30s	84.6	83.2

## Data Availability

The data presented in this study are available on request from the corresponding author.
